# Nonlinear Dynamics of Silicon Nanowire Resonator Considering Nonlocal Effect

**DOI:** 10.1186/s11671-017-2106-9

**Published:** 2017-05-04

**Authors:** Leisheng Jin, Lijie Li

**Affiliations:** 1 School of Electronic Science and Engineering, Nanjing University of Posts and Telecommunications, Nanjing, 210023 China; 2 College of Engineering, Swansea University, Swansea, SA2 8PP UK

**Keywords:** Silicon nanowire resonator, Chaotic vibration, Nonlocal effect

## Abstract

In this work, nonlinear dynamics of silicon nanowire resonator considering nonlocal effect has been investigated. For the first time, dynamical parameters (e.g., resonant frequency, Duffing coefficient, and the damping ratio) that directly influence the nonlinear dynamics of the nanostructure have been derived. Subsequently, by calculating their response with the varied nonlocal coefficient, it is unveiled that the nonlocal effect makes more obvious impacts at the starting range (from zero to a small value), while the impact of nonlocal effect becomes weaker when the nonlocal term reaches to a certain threshold value. Furthermore, to characterize the role played by nonlocal effect in exerting influence on nonlinear behaviors such as bifurcation and chaos (typical phenomena in nonlinear dynamics of nanoscale devices), we have calculated the Lyapunov exponents and bifurcation diagram with and without nonlocal effect, and results shows the nonlocal effect causes the most significant effect as the device is at resonance. This work advances the development of nanowire resonators that are working beyond linear regime.

## Background

Nanoscale resonators working at certain parameters exhibit rich nonlinear dynamics such as chaos and bifurcation [1–4]. To thoroughly investigate the nonlinear dynamics in such nanostructures considering various effects that are brought by reducing the size of the device and/or choosing different fabrication materials is crucial for developing real applications [5–9]. Nonlocal effect, essentially originated from device’s size-reducing, is usually taken into account when studying nanoscale structures in which lattice node interaction is not only affected by its surrounding nodes, but also from the nodes neighboring to the surrounding nodes [10, 11]. This effect has been proved to be playing an important role in nanoscale structures with respect to dynamical response, taking pulling-in as an example [12, 13]. To be more specific, in previous work, nonlocal effects on the elastic behavior of statically bent nanowires have been investigated in [14]. The influences of nonlocal effect on the thermo-electro-mechanical vibration characteristics of piezoelectric nanoplates have been discussed by Chen Liu et al. [15]. Based on a refined nonlocal theory, dynamical behavior of core-shell nanowires with weak interfaces has been analyzed in [16]. Numerically, linear optical response of conducting nanostructures was proved to be altered dramatically by nonlocality [17]. Vibration characteristic of piezoelectric nanobeam under the influence of nonlocal effect has been reported in [18]. However, most of the works so far have been confined in analyzing the nonlocal effect based on linear regime. Even though F. Najar et al. recently investigated the nonlinear static and dynamical response in a nanoactuator taking nonlocal effect into account [19], in which they only studied the puling-in and bulking. How the nonlocal effect exerts its impact on nonlinear dynamics, in particular, the bifurcation and chaos, of nanostructures deserves further investigation.

Here, in this work, we employ silicon nanowire resonator as paradigm and try to systematically derive the expressions linking the nonlocal term with the dynamical parameters such as the resonant frequency, Duffing coefficient, and damping ratio that are directly influence the nonlinear dynamics of the device. Nonlinear dynamics of the resonator is then investigated through the key analysis such as Lyapunov exponent and bifurcation calculation by considering varied nonlocal parameters. Interesting remarks are drawn from the analysis, which in a fundamental way provides a significant result for future device design and modeling and gives useful guidance for the development and design of novel applications based on the nonlinear dynamics of nanowires, e.g., applications such as random generators and secure communications [20].

The report is presented as follows: Model Construction section presents the mathematic derivation of the dynamic equations taking the nonlocal effect into consideration. Numeric response al simulation results are described in Numerical Analysis section. Finally, the key conclusion remarks are summarized in the Conclusions section.

## Methods

**Fig. 1:**
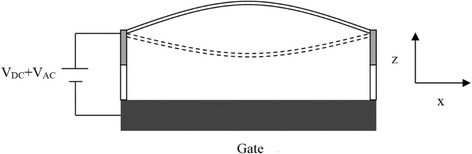
Schematic figure of the double-clamped silicon nanowire resonator

As shown in Fig. 1, the cylindrical double clamped nanowire resonator is driven by electrostatic force exerted between itself and the bottom electrode (also called gate electrode). The driving voltage *V*(*t*) has ac and dc components at the same time, represented by *V*_DC_ and *V*_AC_cos(*ωt*), respectively. *ω* is driving frequency. The nanowire has length *L* and diameter *r*. The material of the nanowire is chosen to be silicon which has density *ρ*, Young’s modulus *E*, and moment of inertia *I*. According to the nonlocal theory proposed in work [21], the nonlocal stress tensor ${\overrightarrow{\sigma}}_N$ is obtained by:

1 $$ {\overrightarrow{\sigma}}_N={\displaystyle \underset{\varOmega}{\int } K\left(\left|{\overrightarrow{x}}^{\prime }-\overrightarrow{x}\right|,\tau \right)}\overrightarrow{\sigma}\left({\overrightarrow{x}}^{\prime}\right) d{\overrightarrow{x}}^{\prime } $$

where $K\left(\left|{\overrightarrow{x}}^{\prime }-\overrightarrow{x}\right|,\tau \right)$ is the nonlocal modulus in which $\left|{\overrightarrow{x}}^{\prime }-\overrightarrow{x}\right|$ is the distance between two points in the lattice. $\overrightarrow{\sigma}\left({\overrightarrow{x}}^{\prime}\right)$ is stress tensor without considering the nonlocal effect.*τ* is the material-depended parameter. In order to implement the nonlocal theory, the authors in [21] have also proposed that Eq. (1) can be equivalently expressed as:

2 $$ \left(1-{\mu}_0^2{\nabla}^2\right){\overrightarrow{\sigma}}_N=\overrightarrow{\sigma},\kern0.5em {\mu}_0={\tau}^2{l}^2={e}_0^2{a}^2 $$

where a and *l* are the internal and external characteristic lengths, *e*_*0*_ is the material constant. By combining the nonlocal theory and beam dynamic theory, the equation that involves the nonlocal effect for describing the motion of the nanowire resonator can be expressed as [5, 19]:

3 $$ \begin{array}{l}\rho A\left(\ddot{W}-{\mu}_0{\ddot{W}}^{{\prime\prime}}\right)+ c\left(\overset{.}{W}-{\mu}_0{\overset{.}{W}}^{{\prime\prime}}\right)-\rho I{\left(\ddot{W}-{\mu}_0{\ddot{W}}^{{\prime\prime}}\right)}^{\prime \prime }+ EI{W^{{\prime\prime}}}^{{\prime\prime} }-\left( T+{T}_0\right){\left( W-{\mu}_0{W}^{{\prime\prime}}\right)}^{\prime \prime }= f-{\mu}_0{f}^{{\prime\prime}}\\ {}\end{array} $$

where *W(x, t)* is the dynamical displacement of the resonator along the x-axis, with the dot and prime are denoting the differentiation with respect to the *t* and *x*, respectively. *T*_0_ and *T* are the initial and induced mechanical tension in the nanowire, respectively.

*A* is the cross-section of the nanowire. *f(x*, *t)* and *c* are the distributed force applied on the nanowire and the damping ratio, respectively, and they have been derived in previous work [4, 22], as:

4 $$ \begin{array}{l} f=-\frac{\pi \varepsilon {V}^2(t)}{\left( Z+ d\right){\left[ \ln \left(4\frac{W\left( x, t\right)+ d}{r}\right)\right]}^2}\\ {}\approx -\frac{2\pi \varepsilon \left({V}_{DC}{V}_{AC}\right) \cos \left(\omega t\right)}{h{\left[ \ln \frac{4 d}{r}\right]}^2}={F}_0 \cos \left(\omega t\right)\end{array} $$

5 $$ c=-\frac{\pi Pd}{4{v}_T} $$

where *ε* is the dielectric constant of the gaseous medium surrounding the resonator. *d* is the initial distance between the nanowire and the gate. In Eq. (4), the approximation has been made based on the assumption that the displacement of the nanowire is much smaller than the gap *d*. In the process of simplification of electric force *f*, we have only kept the harmonic term *2V*_dc_ *V*_ac_cos(*ωt*). The omitted terms in *f* mainly contribute to initial tension, which leads the resonator to a new equilibrium position, while the harmonic term vibrates the resonator and affect its dynamics directly. Meanwhile, other fabrication imperfections would also exist and contribute to the initial tension, resulting in initial stain, which to some extent balance and/or offset the effect brought by omitted terms in *f* [4]. In case that the omitted terms induce a large initial curvature, we, correspondingly, in our model, have incorporated the initial tension *T*_*0*_ to make the dynamical model more reasonable. In Eq. (5), *P* and *T*_*k*_ are the air pressure and temperature, respectively. ${v}_T=\sqrt{k_B{T}_k/ m}$ is the air molecule velocity at *T*_*k*_. *k*_*B*_ is the Boltzmann constant. *m* is the molecular mass of air. In order to conduct the numerical analysis of Eq. (3), Galerkin’s method has been employed [22]. Firstly, the displacement *W*(*x*, *t*) can be written as: *W*(*x*, *t*) *= z*(*t*)*Φ*(*x*), where *Φ*(*x*) *=* (*2*/*3*)^*1*/*2*^[*1-*cos(*2πx*/*L*)] is the deflection eigenmode and *z*(*t*) is the dynamical displacement at the center of the nanowire. *Φ*(*x*) also satisfies the boundary condition *Φ*(0) = *Φ*(*L*) = *Φ*″(0) = *Φ*″(*L*). By substituting *W*(*x*, *t*) *= z*(*t*)*Φ*(*x*) into Eq. (3) and multiplying *Φ*(*x*) on both sides of Eq. (3), then integrating the equation from 0 to *L*, the equation for describing the displacement of the center of the nanowire can be obtained:

6 $$ \begin{array}{l}\left(\rho A\left( L+{\mu}_0\frac{4{\pi}^2}{3 L}\right)+\rho I\left(\frac{4{\pi}^2\left({L}^2+4{\mu}_0{\pi}^2\right)}{3 L{}^3}\right)\right)\ddot{z}\\ {}+ c\left( L+{\mu}_0\frac{4{\pi}^2}{3 L}\right)\overset{.}{z}+\left( EI\left(\frac{16{\pi}^4}{3{L}^3}\right)+{T}_0\left(\frac{4{\pi}^2\left({L}^2+4{\mu}_0{\pi}^2\right)}{3 L{}^3}\right)\right) z\\ {}+\frac{EA}{2 L}\left(\frac{16{\pi}^4\left({L}^2+4{\mu}_0{\pi}^2\right)}{9{L}^4}\right){z}^3=-2\frac{\pi {\varepsilon}_0{V}_{dc}{V}_{ac}}{h{\left( \ln \left(4 h/ d\right)\right)}^2}\left(\sqrt{\frac{2}{3}} L\right) \cos \left(\omega t\right)\end{array} $$

In Eq. (6), it is obvious that the resonate frequency *ω*_*0*_, Duffing coefficient *β*, and damping ratio *γ* are all functions of the nonlocal effect (represented by *μ*_*0*_), and they can be obtained by the following equations:

7 $$ {\omega}_0^2=\frac{\left( EI\left(\frac{16{\pi}^4}{3{L}^3}\right)+{T}_0\left(\frac{4{\pi}^2\left({L}^2+4{\mu}_0{\pi}^2\right)}{3 L{}^3}\right)\right)}{\left(\rho A\left( L+{\mu}_0\frac{4{\pi}^2}{3 L}\right)+\rho I\left(\frac{4{\pi}^2\left({L}^2+4{\mu}_0{\pi}^2\right)}{3 L{}^3}\right)\right)} $$

8 $$ \beta =\frac{\frac{EA}{2 L}\left(\frac{16{\pi}^4\left({L}^2+4{\mu}_0{\pi}^2\right)}{9{L}^4}\right)}{\left(\rho A\left( L+{\mu}_0\frac{4{\pi}^2}{3 L}\right)+\rho I\left(\frac{4{\pi}^2\left({L}^2+4{\mu}_0{\pi}^2\right)}{3 L{}^3}\right)\right)} $$

9 $$ \gamma =\frac{c\left( L+{\mu}_0\frac{4{\pi}^2}{3 L}\right)}{\left(\rho A\left( L+{\mu}_0\frac{4{\pi}^2}{3 L}\right)+\rho I\left(\frac{4{\pi}^2\left({L}^2+4{\mu}_0{\pi}^2\right)}{3 L{}^3}\right)\right)} $$

Eq. (6) can be normalized by using the relations *T = ω*_*0*_ *t* and *Z = z*/*d*, and the normalized equation is expressed as:

10 $$ \ddot{Z}+ Z+\frac{d^2\beta}{{\omega_0}^2}{Z}^3+\frac{\gamma}{\omega_0}\overset{.}{Z}=\frac{F_0}{c_1{d}^2{\omega_0}^2} \cos \left({\omega}_r T\right) $$

where *c*_*1*_is the coefficient of the in Eq. (6) and *ω*_*r*_ *= ω/ω*_*0*_. In the derivation of Eq. (10), the following integrals have been used.

11 $$ \begin{array}{l}{{\displaystyle {\int}_0^L\left({d}^2\varPhi / d{x}^2\right)}}^2 dx=\frac{16{\pi}^4}{3{L}^3};{\displaystyle {\int}_0^L{\varPhi}^2 dx=} L;\\ {}{{\displaystyle {\int}_0^L\left( d\varPhi / d x\right)}}^2 dx=\frac{4{\pi}^2}{3 L};{\displaystyle {\int}_0^L\varPhi} dx=\sqrt{\frac{2}{3}} L\end{array} $$

Based on Eqs. (7), (8), (9), and (10) the dynamics of the nano-resonator can be analyzed.

## Results and discussion

**Fig. 2:**
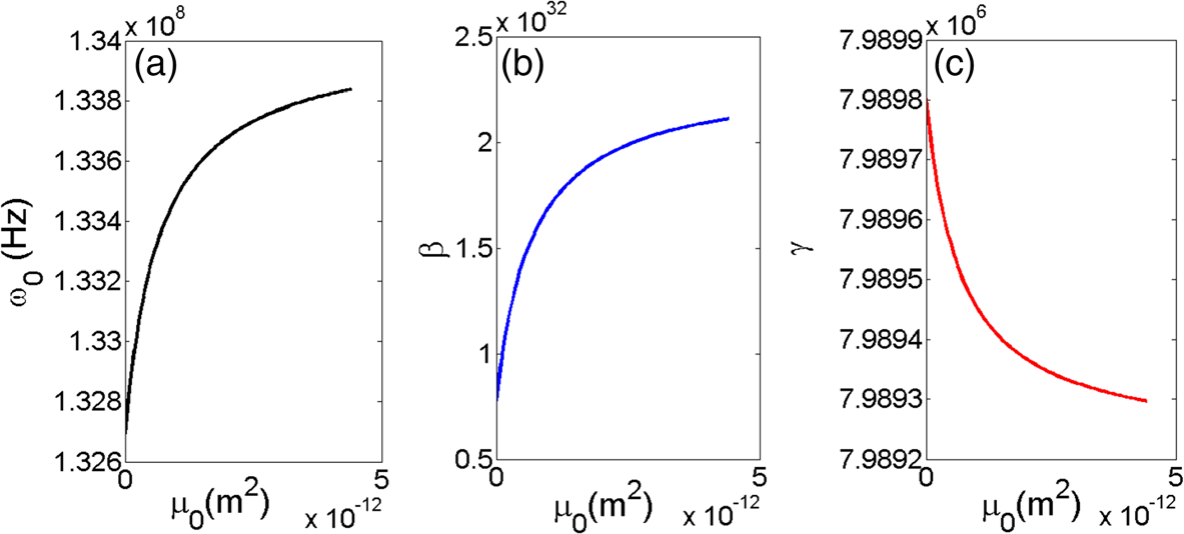
The calculated results of resonate frequency *ω*_0_ (**a**), Duffing coefficient *β* (**b**), and damping ratio *γ* (**c**) related to the nonlocal coefficient *μ*_0_

First of all, based on the derived Eqs. (7)–(9), the relation between the resonant frequency and the nonlocal coefficient has been calculated and shown in Fig. 2a. It is observed that the resonant frequency starts to increase at a small *μ*_*0*_, then increasing trend slows down and the curve tends to reach a saturation point at a very large *μ*_*0*_. Similar trend has been obtained for the relation between the Duffing coefficient and *μ*_*0*_. The result (Fig. 2c) for the damping ratio *γ* in relation to the *μ*_*0*_ is opposite for those demonstrated in Fig. 2a, b, in which starts to fall abruptly in the small *μ*_*0*_ and then gradually approach to a steady point when the *μ*_*0*_ gets large enough. All the results shown in the figure coincide with each other. It is revealed that the resonator responds sensitively with small nonlocal coefficient, and the system reaches to a state that tends to be immune to large nonlocal terms.

**Fig. 3:**
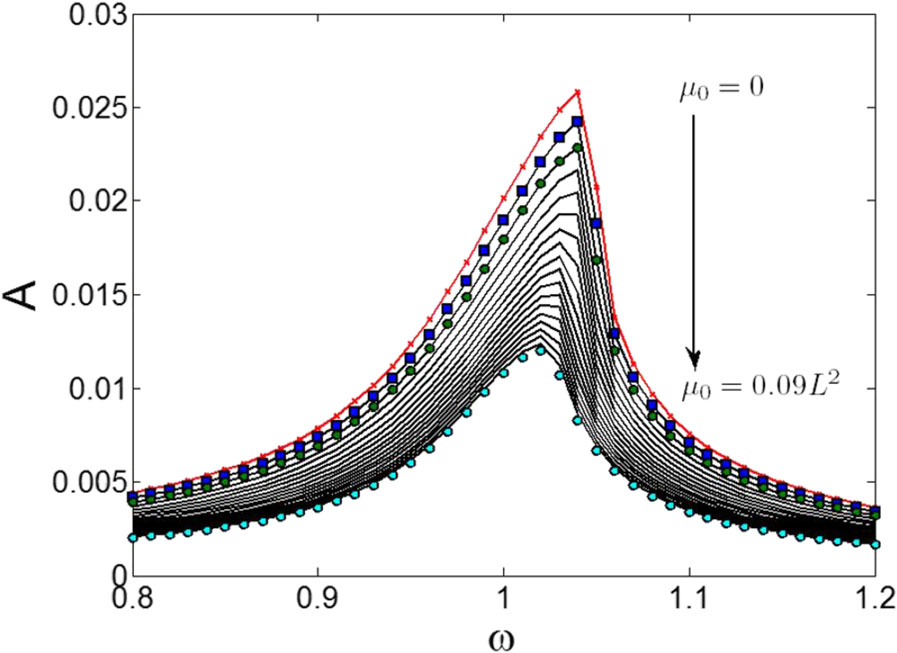
Calculated non-dimensionalised displacement versus the driving frequency of the nanowire resonator

Frequency response of the resonating system has been investigated by taking consideration of the nonlocal term *μ*_*0*_, which is shown in Fig. 3. The displacement amplitude *A* is non-dimensionalized for the middle point of the beam, and the horizontal axis of Fig. 3 is the ratio of the driving signal frequency to the resonant frequency *ω*_*r*_ *= ω/ω*_*0*_ *.* In the result, the peak displacement amplitude reduces from 0.026 to 0.012 as the *μ*_*0*_ increases from 0 to 0.09 *L* ^*2*^, reflecting a more than 50% reduction. The frequency values corresponding to the peak amplitude also reduces from 1.04*ω*_*0*_ to 1.02*ω*_*0*_. It indicates that the nonlocal term effectively hardening the structure and brings the resonator to the more linear state, i.e., the responding frequency is much closer to the modal frequency.

**Fig. 4:**
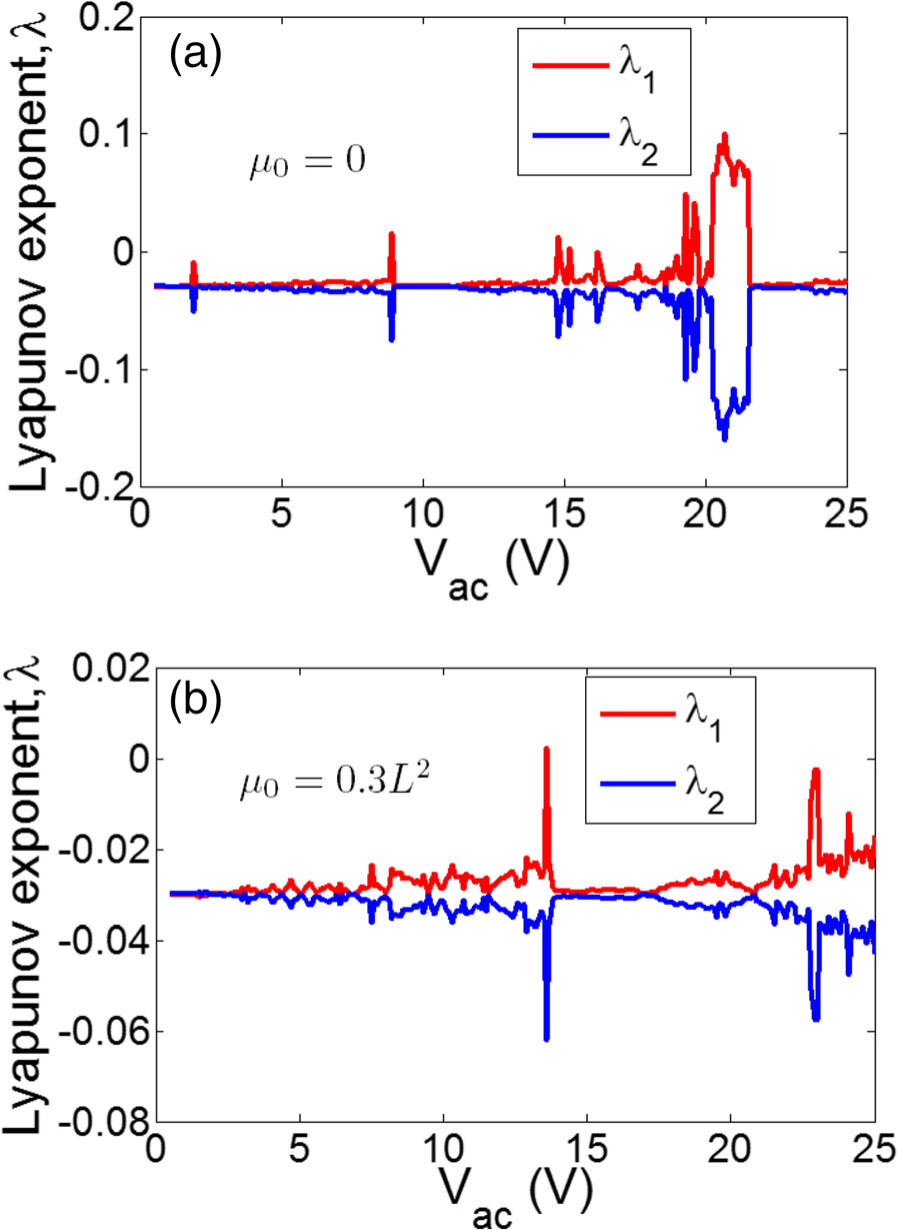
Calcualted Lyapunov exponents (*λ*1 and *λ*2) for Eq. (10). **a** Without nonlocal effect. **b** With nonlocal effect

Figure 4 shows the calculated Lyapunov exponent *λ* of Eq. (10) characterizing the nonlinearity of the structure, in particular, the chaotic vibration behavior. There are two exponents calculated (*λ*_*1*_ and *λ*_*2*_), and only the largest one (*λ*_*1*_) is of importance. Negative *λ* indicates periodical oscillations, and positive *λ* expresses that the system is in chaotic states. It is shown in Fig. 4 that without considering nonlocal effect, under the external ac voltages with the amplitude ranging from around 19 V to 21 V, the resonator experiences chaotic vibrations. However, as a nonlocal term of *μ*_*0*_ = 0.09 L^2^ is imposed, the resonator no longer experiences chaotic oscillations at the same voltage range, indicating effectively hardening effect again, which coincides with the findings shown in Fig. 3.

**Fig. 5:**
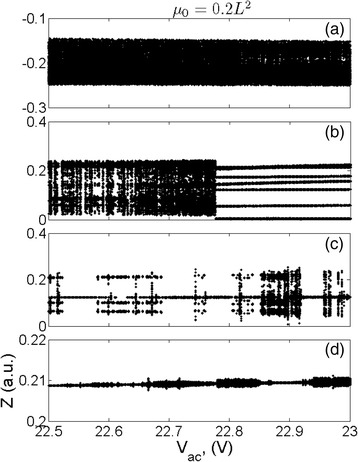
Bifurcation analysis of the resonator without considering the nonlocal effect. **a** The frequency ratio *ω*/*ω*0 is 0.8. **b** The frequency ratio *ω*/*ω*0 is 1. **c** The frequency ratio *ω*/*ω*_0_ is 1.1. **d** The frequency ratio *ω*/*ω*0 is 1.2

**Fig. 6:**
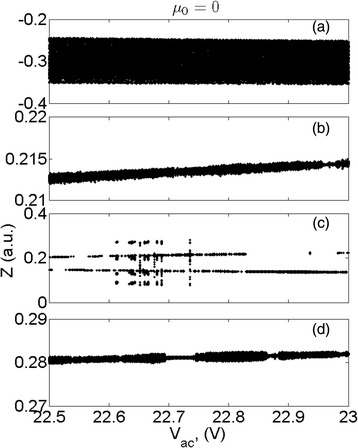
Bifurcation analysis of the resonator considering the nonlocal effect. The frequency ratio *ω*/*ω*0 is 0.8 (**a**), 1 (**b**), 1.1 (**c**), and 1.2 (**d**)

**Fig. 7:**
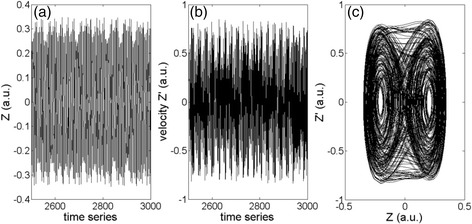
**a** Vibration amplitude versus time. **b** Velocity versus time. **c** Phase portrait. *μ*_0_ = 0, *V*_ac_ = 21 V, and *ω*_1_ = 1

Figures 5 and 6 are results from the bifurcation analysis. It shows (Figs. 5a and 6a) that at the driving frequency is at 0.8*ω*_*0*_, the resonator oscillates similarly for both the cases of considering and neglecting the nonlocal effect. Increasing the driving frequency to *ω*_*0*_, it has been observed in Figs. 5b and 6b that the nonlocal effect makes huge difference on the status of the oscillation. Interestingly, at the case of the considering nonlocal effect, the chaotic vibration has been tuned to few periodical states as the amplitude of the ac voltage in the range of 22.7–22.8 V. Further increasing the driving frequency to 1.1*ω*_*0*_ and 1.2*ω*_*0*_, there is not much difference for both the cases. It unveils that the nonlocal effect mainly impacts on the device vibrating at the region of modal frequency. An example of the device working at chaotic state without considering nonlocal term has been shown in terms of time series and phase portrait in Fig. 7, which matches with the results shown in Fig. 4a.

## Conclusions

Analysis of the nonlinear behavior of the double clamped silicon nanowire resonator with the consideration of the nonlocal effect has been made based on the Duffing motion equation and the Galerkin’s method. Relations between the nonlocal coefficient and dynamic parameters such as resonant frequency, Duffing coefficient, and the damping ratio have been derived. Calculations on the indicator (Lyapunov exponents) of the chaotic vibrations have been conducted, and it is concluded that as the nonlocal term is taken into account, the structure effectively gets hardened and the nonlinear performance has also changed. Importantly, from the bifurcation analysis, the nonlocal effect causes the most significant impact when the driving frequency is at the resonating frequency of the structure. The work provides useful guidance in designing future nanowire resonator-related applications.
